# The trajectory of a range of commonly captured symptoms with standard care in people with kidney failure receiving haemodialysis: consideration for clinical trial design

**DOI:** 10.1186/s12882-023-03394-w

**Published:** 2023-11-17

**Authors:** Pann Ei Hnynn Si, Mónica Hernández-Alava, Louese Dunn, Martin Wilkie, James Fotheringham

**Affiliations:** 1 Sheffield Kidney Institute, Sheffield Teaching Hospitals NHS Foundation Trust, Sheffield, UK; 2 School of Health and Related Research, University of Sheffield, Sheffield, UK

**Keywords:** Haemodialysis, Longitudinal change, PROMS, Symptom burden, Symptom trajectory

## Abstract

**Background:**

Despite the recognized high symptom prevalence in haemodialysis population, how these symptoms change over time and its implications for clinical practice and research is poorly understood.

**Methods:**

Prevalent haemodialysis patients in the SHAREHD trial reported 17 POS-S Renal symptoms (none, mild, moderate, severe and overwhelming) at baseline, 6, 12 and 18 months. To assess the prevalence change at population level in people reporting moderate or worse symptoms at baseline, the absolute change in prevalence was estimated using multi-level mixed effects probit regression adjusting for age, sex, time on haemodialysis and Charlson Comorbidity Score. To assess changes at individual level, the proportion of people changing their symptom score every 6 months was estimated.

**Results:**

Five hundred fifty-two participants completed 1725 questionnaires at four timepoints. Across all 17 symptoms with moderate or worse symptom severity at baseline, the majority of the change in symptom prevalence at population level occurred in the ‘severe’ category. The absolute improvement in prevalence of the ‘severe’ category was ≤ 20% over 18 months in eleven of the seventeen symptoms despite a large degree of relatively balanced movement of individuals in and out of severe category every six months. Examples include depression, skin changes and drowsiness, which had larger proportion (75–80%) moving in and out of severe category each 6 months period but < 5% difference between movement in and out of severe category resulting in relatively static prevalence over time. Meanwhile, larger changes in prevalence of > 20% were observed in six symptoms, driven by a 9 to 18% difference between movement in and movement out of severe category. All symptoms had > 50% of people in severe group changing severity within 6 months.

**Conclusions:**

Changes in the severity of existing symptoms under standard care were frequent, often occurring within six months. Certain symptoms exhibited clinically meaningful shifts at both the population and individual levels. This highlighted the need to consider improvements in symptom severity when determining sample size and statistical power for trials. By accounting for potential symptom improvements with routine care, researchers can design trials capable of robustly detecting genuine treatment effects, distinguishing them from spontaneous changes associated with standard haemodialysis.

**Supplementary Information:**

The online version contains supplementary material available at 10.1186/s12882-023-03394-w.

## Introduction

Globally, the increasing prevalence of kidney failure has resulted in over four million people now requiring kidney replacement therapy (KRT) to sustain life worldwide [1]. Haemodialysis (HD) is the commonest therapy accounting for 69% of all KRT, with standard HD treatment prescription being approximately four hours of HD three times a week [1, 2]. While there have been advancements in haemodialysis treatment, individuals undergoing HD continue to bear a substantial burden of physical and emotional symptoms. These symptoms, as highlighted in prior studies, have been consistently associated with a decline in health-related quality of life (HRQoL), including symptoms such as fatigue, sexual issues, and restless legs having a significant and detrimental impact on HRQoL, further underlining the importance of symptom burden in relation to reduced HRQoL [3–6]. Studies suggested that the impact of symptom burden experienced by people receiving HD may be more important than treatment related clinical parameters in determining the HRQoL in this population [7–9]. Understanding symptom assessment using patient-reported outcome measures (PROMs) should therefore be a fundamental component in the quality of care for people with kidney failure.

Although the cross-sectional prevalence of symptoms in the haemodialysis population is high and well described in the literature, there is limited data on how these symptoms change over time and much of this evidence relates to change in prevalence rather than changes experienced by an individual [10]. There is increasing evidence that symptom burden is the most important predictor of reduced HRQoL amongst people with end staged kidney failure suggesting that recognition and effective treatment of symptom burden may have the greatest impact on the HRQoL in haemodialysis population [11]. In addition to the challenges conducting interventional trials involving HRQoL measures in haemodialysis population, several large randomised clinical trials have failed to demonstrate significant HRQoL advantages from longer or more frequent HD, despite observational data suggesting otherwise [12]. A range of potential explanations for this include failure of existing symptom measures to detect changes in domains which these interventions may modify, and other mechanisms influencing how a patient evaluates their HRQoL: symptoms of chronic disease may change as a result of external factors such as a treatment or a change in health status. Existing literature has found that significant changes in the severity of symptoms occur at a median of 3 months [13] but the degree and direction of change is yet to be explored.

Failure to appreciate how these symptoms change over time in cohorts and individuals receiving haemodialysis for kidney failure threatens the validity of trials of potentially important interventions, preventing their approval or adoption. The primary aim of this study is to assess the change in the prevalence of how individuals undergoing in-centre haemodialysis for kidney failure are affected by symptoms at both the population and individual levels, aiming to provide comprehensive insights into the dynamic nature of symptom experiences in this patient group. The findings of this study are expected to inform the design of future interventional clinical trials, strategically tailored to enhance the HRQoL and looking to reduce the burden of any of the reported symptoms in individuals with kidney failure undergoing haemodialysis.

## Materials and methods

### Study design and setting

This is an observational longitudinal cohort study and secondary analyses from SHAREHD Stepped Wedge Cluster Randomised Trial [14, 15] which evaluated a quality improvement collaborative designed to create an environment to support people with kidney failure receiving in-center HD to dialyze more independently. The evaluation ran for 18 months with an additional six months to assess sustainability, and was conducted across twelve renal centres in England. It ran from October 2016 to October 2018: following a control period of six months. Six centres participated in the intervention immediately with six centres joining after a further six months. The full study protocol and sample size estimation for the primary endpoint are available elsewhere [14].

### Consent, inclusion and exclusion criteria

People established on centre-based HD with capacity to give written informed consent were approached to participate. Inclusion criteria are patients over the age of 18, established on centre-based HD and have capacity to give written informed consent. Exclusion criteria were those who are too unwell to engage in the study, as judged by the clinical team, or unable to understand written and verbal communication in English. Trained, delegated research nurses gained written informed consent to participate from prevalent HD patients established on centre-based haemodialysis. The study adhered to the declaration of Helsinki, ethical approval was obtained from West London & GTAC Research Ethics Committee (IRAS project ID 212395) and the trial was registered (ISRCTN Number 93999549).

### Instruments and data collection

The SHAREHD trial collected The Think Kidneys Your Health Survey (YHS) questionnaires including the POS-S Renal [16]. POS-S renal consists of 17 symptoms commonly experienced by HD patients and each symptom is scored on a five-level ordinal scale: none, mild, moderate, severe, and overwhelming, ranging from zero’none’ to four ‘overwhelming’.

Participants were asked to complete the instruments at baseline, six, 12, and 18 months. A delegated member of the research team collected research nurse completed and self-completed paper instruments, which included demography information (age, gender, ethnicity, and education), comorbidities and HD schedules. The Modified End Stage kidney Disease (ESKD) Charlson comorbidity index (CCI) score [17, 18] was calculated using established algorithms and weights using diagnosis and procedure codes from hospitalisation data obtained through linkage to hospital episode statistics by the National Health Service (NHS) Digital Data Access Request Service.

### Statistical analysis

Participants’ demographic information at baseline was descriptively assessed. Additionally, we conducted descriptive assessments of symptom prevalence at both baseline and during follow-up periods. Baseline characteristics were compared between the cohort who completed the questionnaires at all four timepoints and those who completed one questionnaire. In our data analysis, missing data for the adjustment covariates, specifically comorbidity information, were excluded from consideration. The mechanism for missingness in comorbidity data was attributed to the failure to link this information to the respondent via NHS Digital. Importantly, this missingness was assumed to occur at random.

Respondents reporting moderate or worse for each of these symptoms at baseline were identified as this was analogous to commonly studied clinical trial populations [19]. We performed two main analyses, one examining changes in prevalence at the population level and another studying changes at the individual patient level. For the first analysis, in order to assess the prevalence change at population level, absolute change in prevalence of symptoms were estimated using multi-level mixed effects ordered probit regression adjusting for age (< 40, 40–65, > 65), sex (male and female), time on HD (less than one year, one to five year and more than five year) and Charlson Comorbidity Score (score 0, 1–5 and more than 5), including a quadratic trend term (Additional file 1). Our primary purpose in incorporating these variables is to aid estimation of follow-up observations consistently between routine data collection timepoints and where observations were missing, but we do not assign statistical significance to these predictors. The use of probit mixed effects models allowed the estimation of responses from individuals with missing responses at certain timepoints, under the assumption that the observations are missing at random. Symptoms were then categorized by the absolute improvement in prevalence in the ‘severe’ group, dividing symptoms into two categories to simplify the presentation and understanding of 17 symptoms: symptoms ≤ 20% improvement in prevalence and symptoms with > 20% improvement. The decision to categorize the symptoms into ≦20% and > 20% derived from the aim to distinguish between relatively stable symptoms and less stable symptoms respectively. This binary classification facilitates simpler data interpretation for stakeholders highlighting differences in symptom stability. The choice of threshold was is also informed by the distribution of the data, where a natural break around the 20% threshold is observed, providing statistical support for this categorization. Secondly, to assess changes at an individual level, the proportion of people changing their symptom score (transition probabilities) every 6 months was estimated.

A sensitivity analysis was conducted to test the hypothesis that including all severity of symptoms at baseline (none to overwhelming) may affect the longitudinal changes in this model, fitting the multi-level mixed effects ordered probit regression in participants who reported all severity (none to overwhelming) and none or mild at baseline. All analyses were carried out in STATA version 17.

## Results

### Participants and demographic data

**Fig. 1:**
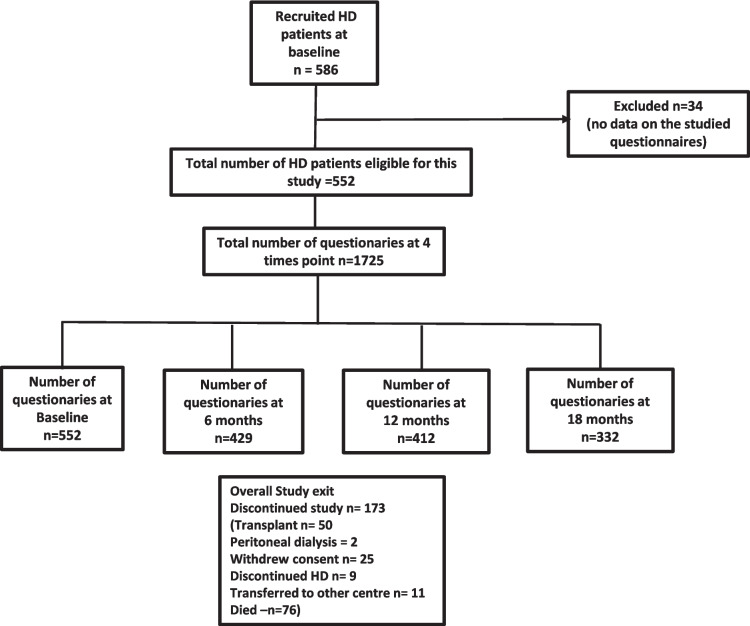
Flow diagram of participants and questionnaires at four time points

Of the 586 participants recruited to the SHAREHD trial, 552 in-centre HD patients from the twelve participating renal centres provided data during the baseline phase, excluding 34 participants with no data on the studied questionnaires. 1725 YHS questionnaires were provided at four times points (552 at baseline, 429 at six months, 412 at twelve and 332 at 18 months) (Fig. 1).

Baseline demographic data of the participants at baseline was described in Table 1. The majority of the participants were male and white, with a mean age of 63 and 5 years on haemodialysis on average at baseline. About one third of the participants had diabetes and quarter had vascular disease. A comprehensive symptom severity sum score ranging from 0 to 68 was calculated for all 17 symptoms at baseline. This score was then divided into three groups based on its distribution. Patient baseline characteristics were subsequently stratified according to their overall symptom severity sum scores, with the groups being: < 13, 13–24, and > 24 (Additional file 2). Participants with highest total symptom severity score were slightly younger and more comorbid (Additional file 2). However, clinical characteristics did not differ significantly between participants who completed instruments at four timepoints and those who completed only one instrument (Additional file 3). Missing items of each symptom at 4 time points were reported as detailed in Additional file 4.

**Table 1:** Participants’ demographic data at baseline

Parameter		Total	Missing
number of participants		552	
Mean Age		63.0 ± 15.6	
Sex (Male)		61.4% (325/529)	4.2% (23/552)
Ethnicity	White	81.6% (427/523)	5.2% (29/552)
Education	No formal education	34.5% (179/519)	6% (33/552)
	High education (1–3)^a^	45.1% (234/519)	
	Higher education (4–6)^b^	20.4% (106/519)	
Myocardial infarction		19.4% (97/501)	
Heart Failure		19.2% (96/501)	
Cerebrovascular accident		7.8% (39/501)	
Diabetes without complication		35.9% (108/501)	
Diabetes with complication		23.2% (116/501)	
Pulmonary Disease		20.8% (104/501)	
Peripheral vascular disease		25.5% (128/501)	
Modified Charlson score index (score 0–16)^c^	Mean score	2.8 ± 2.8	9.2% (51/552)
	Score 0	24.4% (122/501)	
	Score 1–5	61.3% (307/501)	
	Score > 5	14.4% (72/501)	
Years on dialysis	Mean Years on dialysis	5.0 ± 8.0	18.5% (102/552)
	< 1yr	23.3% (105/450)	
	1–5 year	48.7% (219/450)	
	> 5 years	28.0% (126/450)	

Values are given as percentage, mean (± SD), as appropriate

^a^High education (1 = professional qualification, 2 = ’O’ level/GSCE equivalent,3 = Apprenticeship)

^b^Higher education (4 = ’A’ level/higher equivalent,5 = Degree or higher, 6 = Diploma)

^c^Higher Modified Charlson score indicates high comorbidities

### Symptom prevalence at four timepoints

**Fig. 2:**
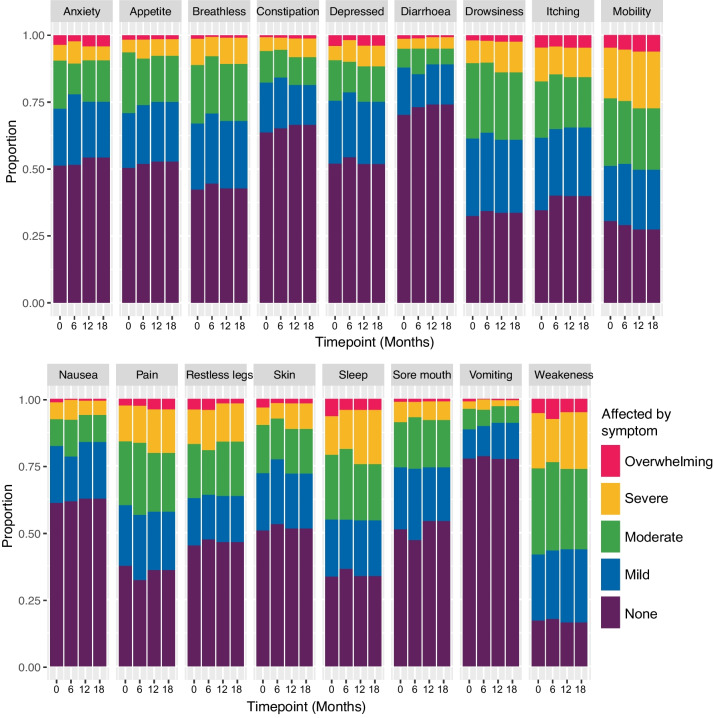
Symptom Prevalence at four timepoints (all severity group from none to overwhelming at baseline). The observations from four time points (baseline, six,12 and 18 months) were used to inform this figure

Overall symptom prevalence at baseline, six, 12 and 18 months is reported in Fig. 2. Each symptom prevalence ranged from the highest prevalence of 80.4% to the lowest of 21.6% at baseline (Fig. 2). On average, participants reported the presence of approximately nine different symptoms (mean 8.9 ± 4.1) at baseline. The most prevalent symptoms at baseline were weakness (80.4%), poor mobility (67.6%), drowsiness (65.4%), difficulty in sleeping (64.6%) and itching (63.1%) (Additional file 5). Among the symptoms reported ‘moderate or worse severity’ at baseline, weakness is the most prevalent, impacting 58.1%, followed by poor mobility at 48.6%, difficult sleeping at 45.0%, and pain at 39.7%. In contrast, less common symptoms at a moderate or worse level include nausea (17.5%), constipation (17.6%), diarrhoea (12.0%), and vomiting (11.4%) as detailed in Additional file 6. The mean number of symptoms reported were similar across time points (mean 8.9 ± 4.1 at baseline, 8.8 ± 4 at six months, 8.7 ± 4 at 12 months and 8.9 ± 4.2 at 18 months).

### Symptoms trajectory in respondents reporting moderate or worse at baseline

**Fig. 3:**
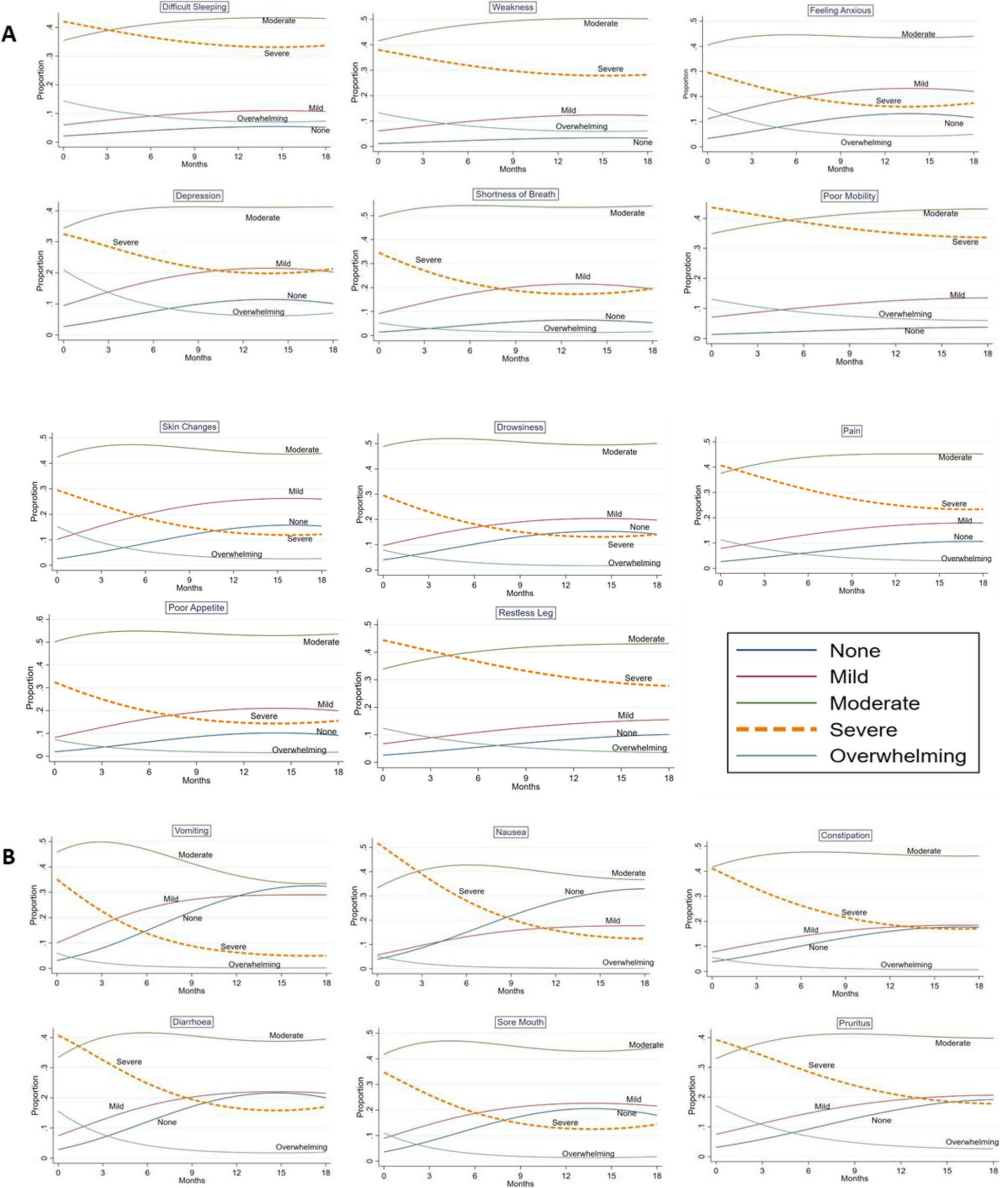
Change in symptoms prevalence over 18 months in people with moderately affected or worse at baseline. This figure was stratified by (**A**) symptoms with ≦20% change and (**B**) > 20% change in the prevalence in those reporting severe degree. Additional files 7 and 8 support this figure

Across all 17 symptoms with moderate or worse symptom severity at baseline, the adjusted multi-level mixed effects ordered probit regression estimated that the majority of the change in population of symptom prevalence occurred in the ‘severe’ category: The prevalence of each symptom generally improved, with reduction in prevalence of the severe category over 18 months while the moderate category remained stable and mild/none prevalence increased (Fig. 3) (Additional files 7 and 8).

**Fig. 4:**
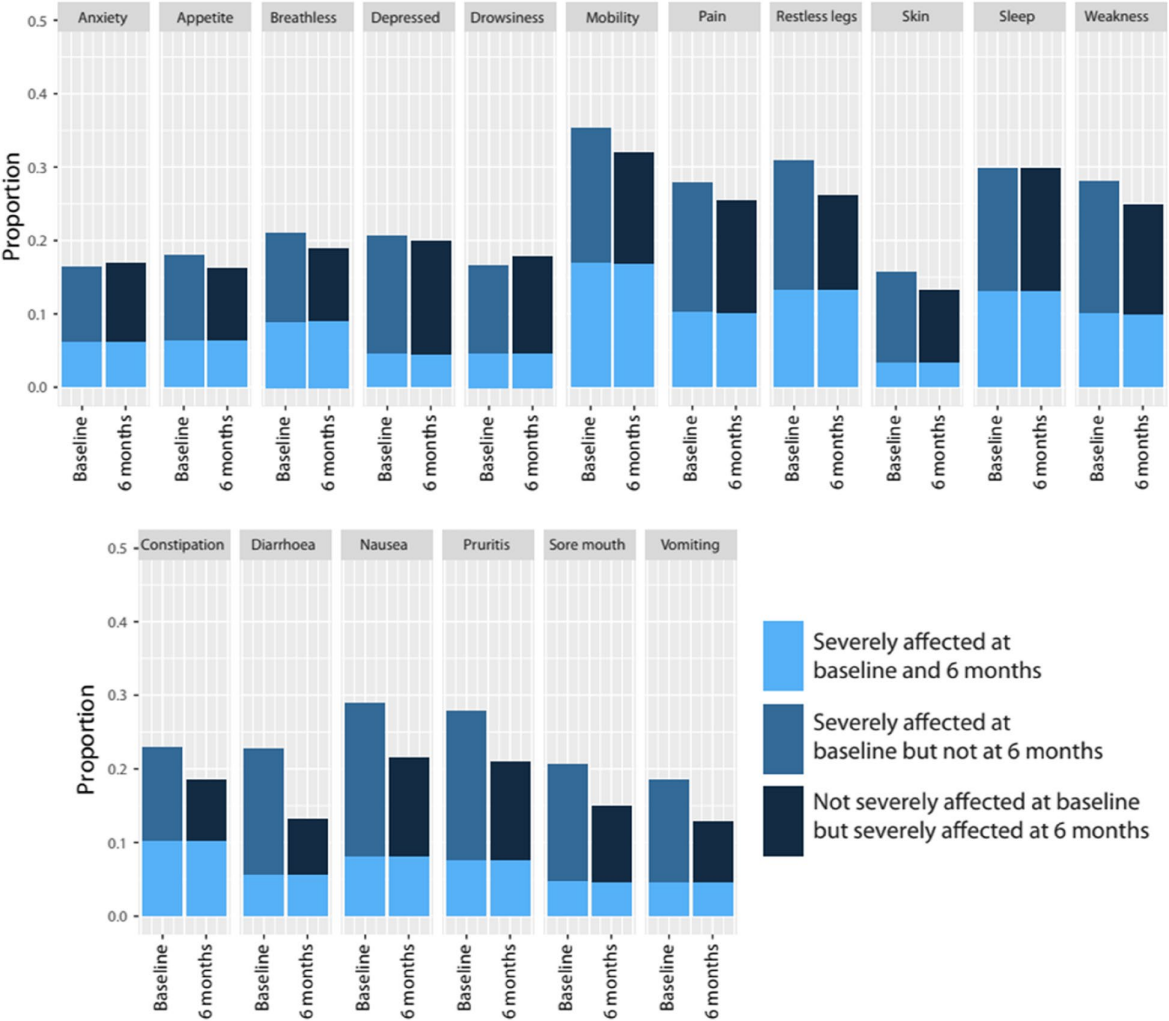
Proportions of people with moderate or worse severity at baseline moving in and out of severe group over 6 months

Eleven symptoms were observed to have a ≤ 20% reduction in the prevalence of the severe category over 18 months indicating more stable symptoms: difficulty sleeping (8.4%), weakness (9.8%), poor mobility (10.0%), depression (11.2%), feeling anxious (12.2%), shortness of breath (15%), drowsiness (15.5%), restless legs (16.6%), poor mobility (16.9%), skin changes (17.3%) and pain (17.3%) (Fig. 3 Panel A) (Additional file 7). The lowest reduction in prevalence (8.4%) was observed in difficulty sleeping (42.1% at baseline, 33.70% at 18 months) whereas the highest change was observed in skin changes and pain (29.5% at baseline, 12.2% at 18 months and 40.7% at baseline, 23.3% at 18 months) (Additional file 7). In order to detect within individual changes, the proportion of people changing their symptom score every six months period in this group was estimated (Fig. 4) (Additional file 9) (Additional file 10 as an example of one symptom). Despite a ≦20% change in the prevalence of severe category for these symptoms, only 20–45% of those reporting severe remained at this level at the next six-monthly questionnaires. This large degree of within-person movement was relatively balanced at a population level by movement in and out of the severe category (Fig. 4) (Additional file 9). For example: only 38.2% of those who reported ‘severe’ degree in feeling anxious at baseline (timepoint zero) remained in the same ‘severe’ degree at six months (timepoint one) with 61.8% moving out of ‘severe’ degree into other categories and while 62.0% of those reporting other categories at baseline had moved into this ‘severe’ category at six months (timepoint one), resulting in 0.2% change in symptom proportions over six months (Additional file 9). Therefore, although there was a large degree of patient movement in and out of ‘severe’ category over six months period, change in proportion of symptom prevalence was minimal. Other examples include depression, skin changes and drowsiness, which had frequent but balanced movement (75%-80%) in and out of severe category over 6 months, leading to a relatively static the prevalence of those with severe symptoms (Additional file 9).

Meanwhile, symptoms with absolute improvements in prevalence of more than 20% were observed in sore mouth (20.3%), pruritus (21.5%), diarrhoea (23.6%), constipation (24.1%), vomiting (30.1%) and nausea (39.5%) (Fig. 3 Panel B) (Additional file 7). Fewer respondents (22–28%) remained in the severe category at any one time over six months period and larger prevalence change was driven by a 9 to 18% difference between the proportion of people moving out of severe compared to those moving into this category (Fig. 4) (Additional file 9). For example: only 25% of those who reported ‘severe’ degree of diarrhoea at baseline (timepoint zero) remained in the same ‘severe’ degree at six months (timepoint one) and 75% moved out into other degrees at six months, while 57% of those who reported ‘severe’ at the six months had moved into this category from other severities at baseline resulting in 18% difference in symptom proportion at six months period (Additional file 9). All symptoms had more than 50% of people in the severe group change their severity over 6 months (Additional file 9).

### Sensitivity analysis

Estimating prevalence on all 17 symptoms with all degrees of severity (from none to overwhelming) at baseline showed little change in symptoms over 18 months period (Additional file 11 showed feeling anxious as an example). In order to test the hypothesis that participants with none or mild symptoms at baseline may not change their symptoms over time, blunting the longitudinal changes in this model, probit regression estimating the trajectory of participants with none or mild symptoms at baseline showed small reduction in none and mild prevalence but moderate or worse remained static over 18 months (Additional file 12).

## Discussion

This longitudinal observational study utilising SHAREHD clinical trial data estimated the trajectory of 17 symptoms informing the POS-S renal questionnaire, and demonstrated a substantial change in symptom burden among people with moderate worse symptoms at baseline, receiving haemodialysis treatment over a six months period. Although differing patterns were identified, improvements in the prevalence of respondents reporting being ‘severely affected’ demonstrated the most notable change over time as more generally symptom severity improves over time. Despite a ≤ 20% change in prevalence of the ‘severe’ category in eleven of the seventeen symptoms, there was still a large degree of within patient movement that was relatively balanced in and out of the severe category. Most importantly, all symptoms had greater than 50% of individuals in the severe group change their severity over 6 months.

Weakness, poor mobility, drowsiness, difficulty in sleeping, and itching were the most commonly reported symptoms, comparable to a number of systematic reviews analysing total symptom burden in this patient group, both in terms of prevalence and type of symptoms reported [20, 21]. Respondent characteristics were comparable to national registry data describing prevalent haemodialysis patients [22]. We demonstrated in sensitivity analyses that including all degrees (none to overwhelming) of symptom severity will lead to symptoms being stable over period. In fact, these have been shown by other studies in a cohort of prevalent HD population: the findings of the study by Davison et [23], a longitudinal study of symptom burden in haemodialysis patients reported no change in mean score of symptoms after 6 months while other studies have showed mixed results [24–26]. It is possible that the reduction in symptom prevalence seen in our study may be a consequence of improved symptom identification and recognition provided by administration of the questionnaire leading to improved symptom management. This phenomenon should affect all longitudinal symptom questionnaire studies where the clinical team observe responses and would argue that routine measurement of PROMs in clinical setting may potentially help improve symptom burden in haemodialysis populations. Our study revealed minimal variations over time for individuals with none to mild symptoms. In contrast, the 'severe' group exhibited significant shifts in prevalence. This dynamic could be attributed to individuals with 'severe' symptoms actively seeking assistance or being recognized by healthcare professionals. Optimizing standard haemodialysis therapy may contribute to the observed improvement in this group.

This study has several strengths. This study explored the longitudinal change in symptoms over time as most previous studies assessing symptom burden in patients with advanced chronic kidney disease did so cross-sectionally. Strengths include the reporting of a representative and diverse cohort of haemodialysis patients and use of a symptom assessment tool that has been validated in the HD population. In 62 participants, follow up questionnaires were missing. By using probit mixed effects models, we were able to analyse data from individuals where responses at some timepoints are missing, assumed at random. There are some limitations to this study. We have not analysed how symptom severity may be associated with change in therapy (pharmacological, change in HD frequency) and acute illness as these were not captured, however these issues would occur often in research settings and routinely in clinical settings. The majority of the questionnaires were completed during HD treatment meaning any impact of the timing of completion cannot be assessed although we have reported that that symptoms burden is not affected by HD day of the week [27].

There was lack of evidence on how frequently these PROMs should be measured and the impact of recruiting people with certain severity of symptoms in clinical trials [28]. As over half of individuals with severe symptom burden can change their symptom severity within 6 months, we argue for routine and frequent measurement of symptom-based PROMs in haemodialysis populations to identify individuals requiring intervention. We recommend considering that if people with severe severity at baseline are recruited, spontaneous improvement could be expected. The recognition of such improvement should prompt a thorough examination of current standard care protocols and creates opportunities for targeted interventions. Research is needed to identify the clinical practices that have led to improvements in individuals severely affected by symptoms which could be evaluated in clinical trials. Moreover, for clinical trials enrolling populations with moderate or worse symptoms, the presented data can be utilized to understand the proportions improving under standard care. If a run-in period is deemed necessary, this analysis can guide decisions on this period’s duration and the proportion of participants that might be excluded.

Insights gained from the observed improvement under standard care may guide the selection of relevant outcome measures and the determination of appropriate follow-up duration. Overall, acknowledging and investigating the observed symptom improvement within the context of standard care lays a foundation for refining and optimizing clinical trial designs focused on improving the health-related quality of life for haemodialysis patients.

## Conclusion

We observed a substantial burden of symptoms in a diverse and representative, prospective haemodialysis cohort. A change in the severity of existing symptoms in response to standard haemodialysis care was very common and can occur within six months. Considering clinically meaningful changes at the population and patient level in some symptoms, this highlights the importance of accounting for natural variations or improvements in symptoms when determining the sample size and statistical power of a trial. By taking into consideration the potential for improvement with routine care, researchers can design trials that are robust enough to detect true treatment effects, distinguishing them from changes that might occur spontaneously in response to standard HD.

### Supplementary Information

**Additional file 1. **Quadratic probit regression model estimating the probabilities (Example of probit regression output for one symptom (Nausea)).

**Additional file 2. **Demographic data stratified by total symptom severity score at baseline.

**Additional file 3. **Baseline demographic comparison between participants with no follow up and completed follow up.

**Additional file 4. **Missing item of POS S renal questionnaires.

**Additional file 5. **Symptoms prevalence at baseline (the presence of symptoms from mild to overwhelming).

**Additional file 6. **Prevalence of moderate or worse (moderate, severe, and overwhelming) severity at baseline.

**Additional file 7. **Proportion of prevalence change in ‘SEVERE’ category over 18 months in people with moderate or worse at baseline.

**Additional file 8. **Proportion of prevalence change in ‘other categories’ (none, mild, moderate, overwhelming) over 18 months in people with moderate or worse at baseline.

**Additional file 9. **Proportions /probabilities of people with moderate or worse severity at baseline moving in and out of severe group over 6 months.

**Additional file 10. **Proportion of change in symptom (feeling anxious) over 6 months period.

**Additional file 11. **Example of symptom trajectory of feeling anxious (all degrees of severity at baseline).

**Additional file 12. **Example of symptom trajectory of feeling anxious (none or mild at baseline).

## Data Availability

A minimal dataset required to reach the conclusions drawn from this manuscript required the linkage of identifiable patient information collected during the trial to Hospital Episode Statistics data, which at the time of writing is provided by the NHS Digital Data Access Request Service (NHS DARS, https://digital.nhs.uk/services/data-access-request-service-dars), and then appropriate processing. An application to NHS DARS can be submitted detailing lawful processing of the combined dataset and the period which HES data is required for. NHS DARS would verify appropriate permissions were in place as a result of this process. A data sharing agreement between the relevant parties would allow data to be transferred from the University of Sheffield to NHS DARS and on to those wishing to perform the enclosed analyses. Please contact ctru@sheffield.ac.uk for further information about the unlinked dataset which has the personal information required for linkage.

## Abbreviations

PROMS: Patient reported outcome measures
HRQoL: Health-related quality of life
KRT: Kidney replacement therapy
HD: Haemodialysis
YHS: The Think Kidneys Your Health Survey
