# Supplementary material for: The trajectory of a range of commonly captured symptoms with standard care in people with kidney failure receiving haemodialysis: consideration for clinical trial design
Source: BMC Nephrol. 2023 Nov 17;24:341. doi: 10.1186/s12882-023-03394-w (PMC10656962; doi:10.1186/s12882-023-03394-w)
Supplement: Supplementary file 1 — Additional file 1. Quadratic probit regression model estimating the probabilities (Example of probit regression output for one symptom (Nausea)). [file 12882_2023_3394_MOESM1_ESM.docx]

**Additional file 1: Quadratic trend probit regression model estimating the probabilities (Example of probit regression output for one symptom (Nausea))**

This Stata code performs a mixed-effects ordinal probit regression (meoprobit) to model the relationship between the dependent variable YSQ4 (nausea in this code) and various predictor variables (age, sex, vintage and co morbidities), including a quadratic trend term. The model is estimated with fixed effects and grouped by the SHAREHD_ID variable. The estimated coefficients for the fixed effects and cut points are presented, indicating the impact of each predictor on the odds of the ordinal outcomes. The model generates predicted probabilities (prqua*) based on fixed effects and posterior means of random effects. After summarizing the predicted probabilities, the code enters a loop to calculate predicted probabilities for different time point(weekly which is 79 weeks in total , equivalent of 18 months) . (r1 represents each week , prop 1 = none, prop 2= mild, prop 3= moderate, prop 4= severe , prob 5= overwhelming) . These probabilities are stored in matrices, and the final results are displayed as a list. The code then restores the original dataset and creates a line plot depicting the relationship between the predicted probabilities and weeks.

This Stata code and output examines the distribution and transformation of variables within a panel dataset. This conducts a panel data analysis, initially setting up the data for panel structure and subsequently examining the distribution of variable (Nausea) within this panel using the xttrans command. The analysis aims to understand patterns and frequencies in the dataset across different entities and time points.
