# Supplementary material for: The trajectory of a range of commonly captured symptoms with standard care in people with kidney failure receiving haemodialysis: consideration for clinical trial design
Source: BMC Nephrol. 2023 Nov 17;24:341. doi: 10.1186/s12882-023-03394-w (PMC10656962; doi:10.1186/s12882-023-03394-w)
Supplement: Supplementary file 2 — Additional file 2. Demographic data stratified by total symptom severity score at baseline. [file 12882_2023_3394_MOESM2_ESM.docx]

**Table 1: Demographic data stratified by total symptom severity score at baseline**

| Parameter | | Total symptom severity score <13 | Total symptom severity score 13-24 | Total symptom severity score >24 | Total |
| --- | --- | --- | --- | --- | --- |
| number of participants | | 34% (192/552) | 33% (182/552) | 32.2% (178/552) | 552 |
| Mean Age | | 64.3±15.5 | 63.0±15.9 | 61.5±15.2 | 63.0±15.6 |
| Sex (Male) | | 63.1% (118/187) | 63.8% (111/174) | 57.1% (96/168) | 61.4% (325/529) |
| Ethnicity | White | 79.8% (146/183) | 83.9% (146/174) | 81.3% (135/166) | 81.6% (427/523) |
| Education | No formal education | 28.8% (53/184) | 38.5% (65/169) | 36.7% (61/166) | 34.5% (179/519) |
|  | High education (1-3)* | 45.1% (83/184) | 46.7% (79/169) | 43.4% (72/166) | 45.1% (234/519) |
|  | Higher education (4-6)** | 26.1% 48/184) | 14.8% (25/169) | 19.9% (33/166) | 20.4% (106/519) |
| Myocardial infarction | | 15.8% (28/177) | 19.6% (31/158) | 22.9% (38/166) | 19.4% (97/501) |
| Heart Failure | | 12.4% (22/177) | 24.7% (39/158) | 21.1% (35/166) | 19.2% (96/501) |
| Cerebrovascular accident | | 5.1% (9/177) | 8.9% (14/158) | 9.6% (16/166) | 7.8% (39/501) |
| Diabetes without complication | | 32.2% (57/177) | 38.0% (60/158) | 38.0% (63/166) | 35.9% (108/501) |
| Diabetes with complication | | 18.6% (33/177) | 28.5% (45/158) | 22.9% (38/166) | 23.2% (116/501) |
| Pulmonary Disease | | 11.9% (21/177) | 22.8% (36/158) | 28.3% (47/166) | 20.8% (104/501) |
| Peripheral vascular disease | | 24.9% (44/177) | 29.1% (46/158) | 22.9% (38/166) | 25.5% (128/501) |
| Modified Charlson score index (score 0-16)*** | Mean score | 2.4±2.6 | 3.1±2.9 | 3.0±2.8 | 2.8±2.8 |
|  | Score 0 | 26.6% (47/177) | 22.8% (36/158) | 23.5% (39/166) | 24.4% (122/501) |
|  | Score 1-5 | 64.4% (114/177) | 62.0% (98/158) | 57.2% (95/166) | 61.3% (307/501) |
|  | Score >5 | 9.0% (16/177) | 15.2% (24/158) | 19.3% (32/166) | 14.4% (72/501) |
| Years on dialysis | Mean Years on dialysis | 4.9 ± 6.5 | 5.0 ± 7.5 | 5.0 ± 10.1 | 5.0±8.0 |
|  | <1yr | 22.8% (38/167) | 25.0% (36/144) | 22.3% (31/139) | 23.3% (105/450) |
|  | 1-5 year | 47.3% (79/167) | 47.2% (68/144) | 51.8% (72/139) | 48.7% (219/450) |
|  | >5 years | 29.9% (50/167) | 27.8% (40/144) | 25.9% (36/139) | 28.0% (126/450) |

**Values are given as percentage, mean (±SD), as appropriate.**

**Total symptom severity score was categorised into 3 groups based on their distribution.**

***High education (1=professional qualification, 2=’O’ level/GSCE equivalent,3=Apprenticeship)**

****Higher education (4=’A’ level/higher equivalent,5=Degree or higher, 6=Diploma)**

***** Higher Modified Charlson score indicates high comorbidities.**
