# Supplementary material for: The trajectory of a range of commonly captured symptoms with standard care in people with kidney failure receiving haemodialysis: consideration for clinical trial design
Source: BMC Nephrol. 2023 Nov 17;24:341. doi: 10.1186/s12882-023-03394-w (PMC10656962; doi:10.1186/s12882-023-03394-w)
Supplement: Supplementary file 3 — Additional file 3. Baseline demographic comparison between participants with no follow up and completed follow up. [file 12882_2023_3394_MOESM3_ESM.docx]

**Additional File 3: Baseline demographic comparison between participants with no follow up and completed follow up**

| Parameter | | Completed all 4 observations | No follow up observations | P value |
| --- | --- | --- | --- | --- |
| Participants number | | 280 | 62 |  |
| Mean Age | | 64.1+/-14.9 | 64.8 +/- 16.1 | 0.732 |
| Sex (Male) | | 66.3% (185/279) | 64.2% (34/53) | 0.76 |
| Ethnicity | White | 83.3% (229/275) | 81.3% (43/53) |  |
| Education | No formal education | 35.1% (95/271) | 41.2% (21/51) |  |
|  | High education (1-3) | 43.5% (118/271) | 45.1% (23/51) |  |
|  | Higher education (4-6) | 21.4% (58/271) | 13.7% (7/51) |  |
| Myocardial infarction | | 18.9% (48/254) | 16.7% (9/54) |  |
| Heart Failure | | 17.3% (44/254) | 22.2% (12/54) |  |
| CVA | | 7.5% (19/254) | 11.1% (6/54) |  |
| DM without complication | | 36.2% (92/254) | 33.3% (18/54) |  |
| DM with complication | | 22.8% (58/254) | 14.8% (8/54) |  |
| Pulmonary Disease | | 18.9% (48/254) | 18.5% (10/54) |  |
| PVD | | 25.6% (65/254) | 25.9% (14/54) |  |
| Modified Charlson score index (score 0-16) | Score 0 | 20.5% (52/254) | 27.8% (15/54) |  |
|  | Score 1-5 | 65.7% (167/254) | 63.0% (34/54) |  |
|  | Score >5 | 13.8% (35/254) | 9.3% (5/54) |  |
| Years on dialysis | Mean Years on dialysis | 5.8+/-9.0 | 5.4+/-8.8 | 0.836 |
|  | <1yr on RRT | 23.2% (57/246) | 28.9% (13/45) |  |
|  | 1-5 year | 49.2% (121/246) | 40% (18/45) |  |
|  | >5 years | 27.6% (68/246) | 31.1% (14/45) |  |

**Chi square test and ANOVA were used to compare these two cohorts of participants.**
