# Supplementary material for: The trajectory of a range of commonly captured symptoms with standard care in people with kidney failure receiving haemodialysis: consideration for clinical trial design
Source: BMC Nephrol. 2023 Nov 17;24:341. doi: 10.1186/s12882-023-03394-w (PMC10656962; doi:10.1186/s12882-023-03394-w)
Supplement: Supplementary file 4 — Additional file 4. Missing item of POS S renal questionnaires. [file 12882_2023_3394_MOESM4_ESM.docx]

Additional file 4 : Missing item of POS S renal questionnaires

| **Symptoms** | **Baseline** | **6 months** | **12 months** | **18 months** | **Total** |
| --- | --- | --- | --- | --- | --- |
| **difficult sleeping** | 2.7% (15/552) | 0.2% (1/429) | 1.5% (6/412) | 1.2% (4/332) | **1.5% (26/1725)** |
| **weakness** | 2.9% (16/552) | 1.2% (5/429) | 2.4% (10/412) | 1.8% (6/332) | **2.2% (37/1725)** |
| **Feeling anxious** | 2.9% (16/552) | 0.5% (2/429) | 1.2% (5/412) | 0.3% (1/332) | **1.4% (24/1725)** |
| **depression** | 3.1% (17/552) | 0.5% (2/429) | 1.2% (5/412) | 0.3% (1/332) | **1.5% (25/1725)** |
| **Shortness of breath** | 3.8% (21/552) | 0.9% (4/429) | 2.2% (9/412) | 1.2% (4/332) | **2.2% (38/1725)** |
| **poor mobility** | 2.7% (15/552) | 1.2% (5/429) | 1.5% (6/412) | 0.3% (1/332) | **1.6% (27/1725)** |
| **change in skin** | 3.8% (21/552) | 0.9% (4/429) | 2.4% (10/412) | 0.6% (2/332) | **2.2% (37/1725)** |
| **drowsiness** | 3.3% (18/552) | 1.4% (6/429) | 1.7% (7/412) | 0.6% (2/332) | **1.9% (33/1725)** |
| **pain** | 3.6% (20/552) | 1.6% (7/429) | 1.5% (6/412) | 0.6% (2/332) | **2% (35/1725)** |
| **poor appetite** | 3.1% (17/552) | 0.7% (3/429) | 1.5% (6/412) | 0.3% (1/332) | **1.6% (27/1725)** |
| **restless legs** | 3.5% (19/552) | 0.2% (1/429) | 2.2% (9/412) | 0.3% (1/332) | **1.7% (30/1725)** |
| **Vomiting** | 3.1% (17/552) | 0.9% (4/429) | 1.7% (7/412) | 2.4% (8/332) | **2.1% (36/1725)** |
| **Nausea** | 3.3% (18/552) | 1.4% (6/429) | 1.9% (8/412) | 1.2% (4/332) | **2.1% (36/1725)** |
| **constipation** | 3.6% (20/552) | 0.9% (4/429) | 1.7% (7/412) | 0.6% (2/332) | **1.9% (33/1725)** |
| **diarrhoea** | 3.6% (20/552) | 0.5% (2/429) | 1.7% (7/412) | 0.9% (3/332) | **1.9% (32/1725)** |
| **Sore mouth** | 2.9% (16/552) | 0.7% (3/429) | 1.2% (5/412) | 0.3% (1/332) | **1.5%(25/1725)** |
| **pruritis** | 3.5% (19/552) | 0.9% (4/429) | 1.9% (8/412) | 0.6% (2/332) | **1.9% (33/1725)** |
