# Supplementary material for: The trajectory of a range of commonly captured symptoms with standard care in people with kidney failure receiving haemodialysis: consideration for clinical trial design
Source: BMC Nephrol. 2023 Nov 17;24:341. doi: 10.1186/s12882-023-03394-w (PMC10656962; doi:10.1186/s12882-023-03394-w)

Additional file 5: Symptoms prevalence at baseline (the presence of symptoms from mild to overwhelming)


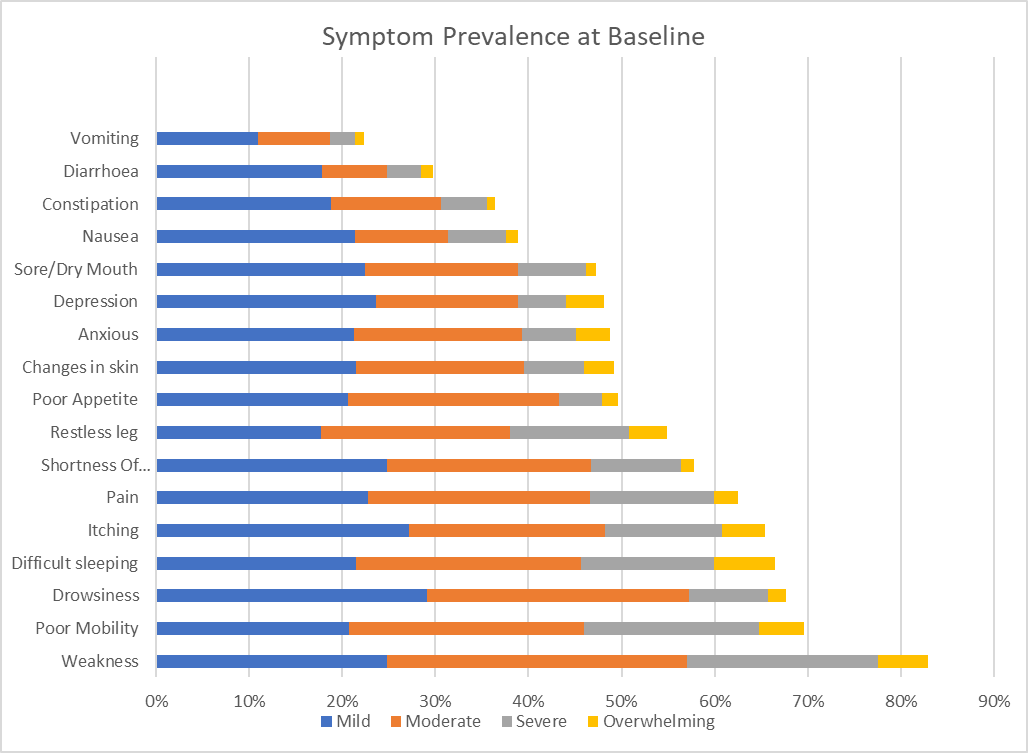

Supplement: Supplementary file 5 — Additional file 5. Symptoms prevalence at baseline (the presence of symptoms from mild to overwhelming). [file 12882_2023_3394_MOESM5_ESM.docx]
