# Supplementary material for: The trajectory of a range of commonly captured symptoms with standard care in people with kidney failure receiving haemodialysis: consideration for clinical trial design
Source: BMC Nephrol. 2023 Nov 17;24:341. doi: 10.1186/s12882-023-03394-w (PMC10656962; doi:10.1186/s12882-023-03394-w)

Additional file 6: Prevalence of moderate or worse (moderate, severe and overwhelming) severity at baseline


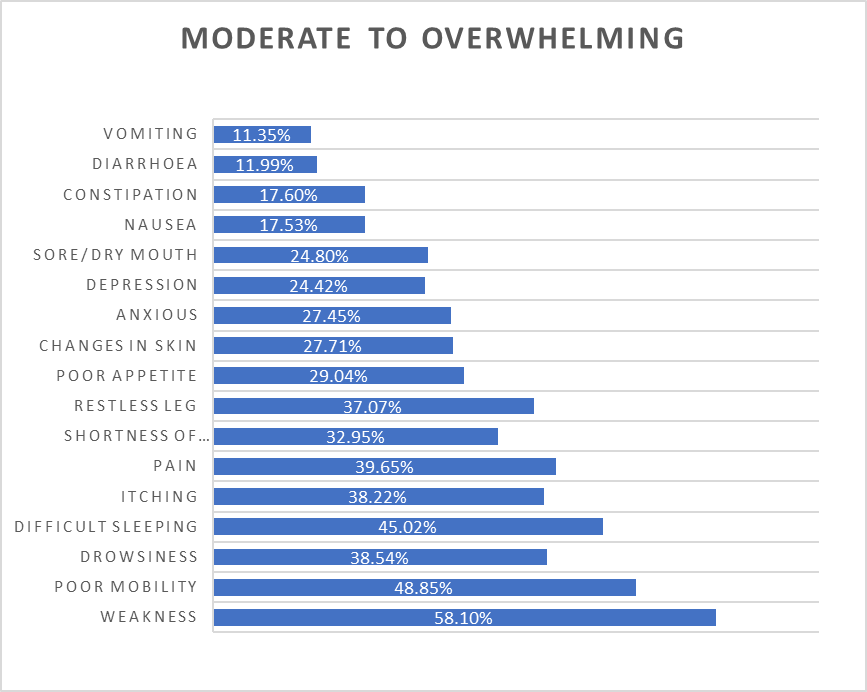

Supplement: Supplementary file 6 — Additional file 6. Prevalence of moderate or worse (moderate, severe, and overwhelming) severity at baseline. [file 12882_2023_3394_MOESM6_ESM.docx]
