# Supplementary material for: The trajectory of a range of commonly captured symptoms with standard care in people with kidney failure receiving haemodialysis: consideration for clinical trial design
Source: BMC Nephrol. 2023 Nov 17;24:341. doi: 10.1186/s12882-023-03394-w (PMC10656962; doi:10.1186/s12882-023-03394-w)
Supplement: Supplementary file 7 — Additional file 7. Proportion of prevalence change in ‘SEVERE’ category over 18 months in people with moderate or worse at baseline. [file 12882_2023_3394_MOESM7_ESM.docx]

**Additional File 7: Proportion of prevalence change in ‘SEVERE’ category over 18 months in people with moderate or worse at baseline**

| **‘Severe’ Symptoms** | **Population prevalence at baseline** | **Population prevalence at 6 months** | **Population prevalence at 12 months** | **Population prevalence at 18 months** | **Change in population prevalence over 18 months period** | |
| --- | --- | --- | --- | --- | --- | --- |
| **difficult sleeping** | 42.1% | 36.8% | 33.5% | 33.7% | **8.4%** | **≦20% change** |
| **weakness** | 38.0% | 32.1% | 28.4% | 28.2% | **9.8%** |  |
| **Feeling anxious** | 29.6% | 20.6% | 16.2% | 17.4% | **12.2%** |  |
| **depression** | 32.5% | 24.7% | 20.1% | 21.3% | **11.2%** |  |
| **Shortness of breath** | 34.6% | 22.2% | 17.4% | 19.6% | **15.0%** |  |
| **poor mobility** | 43.7% | 38.8% | 35.1% | 33.6% | **10.0%** |  |
| **change in skin** | 29.5% | 18.9% | 13.0% | 12.2% | **17.3%** |  |
| **drowsiness** | 29.6% | 18.5% | 13.5% | 14.1% | **15.5%** |  |
| **pain** | 40.7% | 31.4% | 25.1% | 23.3% | **17.3%** |  |
| **poor appetite** | 32.4% | 20.0% | 14.7% | 15.6% | **16.9%** |  |
| **restless legs** | 44.4% | 36.8% | 30.7% | 27.8% | **16.6%** |  |
| **Vomiting** | 35.1% | 14.4% | 6.4% | 5.0% | **30.1%** | **>20% change** |
| **Nausea** | 51.8% | 28.8% | 16.0% | 12.4% | **39.5%** |  |
| **constipation** | 41.2% | 26.8% | 18.67% | 17.1% | **24.1%** |  |
| **diarrhoea** | 40.7% | 25.3% | 16.8% | 17.0% | **23.6%** |  |
| **Sore mouth** | 34.7% | 19.4% | 12.89% | 14.4% | **20.3%** |  |
| **pruritis** | 39.3% | 28.9% | 20.8% | 17.8% | **21.5%** |  |
