# Supplementary material for: The trajectory of a range of commonly captured symptoms with standard care in people with kidney failure receiving haemodialysis: consideration for clinical trial design
Source: BMC Nephrol. 2023 Nov 17;24:341. doi: 10.1186/s12882-023-03394-w (PMC10656962; doi:10.1186/s12882-023-03394-w)
Supplement: Supplementary file 8 — Additional file 8. Proportion of prevalence change in ‘other categories’ (none, mild, moderate, overwhelming) over 18 months in people with moderate or worse at baseline. [file 12882_2023_3394_MOESM8_ESM.docx]

**Additional File 8: Proportion of prevalence change in ‘other categories’ (none, mild, moderate, overwhelming) over 18 months in people with moderate or worse at baseline**

**(The severe category was reported separately in Additional File 7, as it constituted the primary area of interest in this study.)**

| **Symptoms** |  | **Population prevalence at baseline** | **Population prevalence at 6 months** | **Population prevalence at 12 months** | **Population prevalence at 18 months** |
| --- | --- | --- | --- | --- | --- |
| **difficult sleeping** | None | 2.1% | 3.9% | 5.3% | 5.2% |
|  | Mild | 6.0% | 9.0% | 10.7% | 10.6% |
|  | Moderate | 35.4% | 41.1% | 43.2% | 43.1% |
|  | Overwhelming | 14.4% | 9.3% | 7.2% | 7.3% |
| **weakness** | None | 1.1% | 2.3% | 3.2% | 3.3% |
|  | Mild | 6.1% | 9.7% | 12.0% | 12.2% |
|  | Moderate | 41.5% | 47.8% | 50.1% | 50.2% |
|  | Overwhelming | 13.3% | 8.2% | 6.2% | 6.1% |
| **Feeling anxious** | None | 3.3% | 8.8% | 12.9% | 11.6% |
|  | Mild | 11.1% | 19.2% | 23.0% | 22.0% |
|  | Moderate | 40.5% | 44.6% | 43.5% | 44.0% |
|  | Overwhelming | 15.5% | 6.8% | 4.3% | 4.9% |
| **depression** | None | 2.7% | 7.4% | 11.2% | 10.1% |
|  | Mild | 9.4% | 17.3% | 21.3% | 20.2% |
|  | Moderate | 34.4% | 40.6% | 41.2% | 41.3% |
|  | Overwhelming | 21.0% | 9.7% | 6.3% | 7.1% |
| **Shortness of breath** | None | 1.4% | 4.3% | 6.4% | 5.3% |
|  | Mild | 9.2% | 17.3% | 21.4% | 19.4% |
|  | Moderate | 49.5% | 54.2% | 53.5% | 54.0% |
|  | Overwhelming | 5.3% | 2.1% | 1.3% | 1.6% |
| **poor mobility** | None | 1.3% | 2.3% | 3.3% | 3.8% |
|  | Mild | 7.1% | 10.1% | 12.5% | 13.5% |
|  | Moderate | 34.9% | 39.8% | 42.4% | 43.1% |
|  | Overwhelming | 13.1% | 8.8% | 6.7% | 6.0% |
| **change in skin** | None | 2.6% | 8.3% | 14.3% | 15.4% |
|  | Mild | 10.2% | 19.8% | 25.3% | 26.0% |
|  | Moderate | 42.5% | 47.2% | 44.6% | 43.8% |
|  | Overwhelming | 15.2% | 5.7% | 2.9% | 2.6% |
| **drowsiness** | None | 4.0% | 10.0% | 14.8% | 14.2% |
|  | Mild | 9.7% | 16.6% | 20.1% | 19.7% |
|  | Moderate | 48.9% | 51.9% | 49.8% | 50.2% |
|  | Overwhelming | 7.9% | 3.0% | 1.7% | 1.9% |
| **pain** | None | 2.6% | 6.0% | 9.4% | 10.6% |
|  | Mild | 7.8% | 13.2% | 16.9% | 17.9% |
|  | Moderate | 37.5% | 43.6% | 45.2% | 45.2% |
|  | Overwhelming | 11.3% | 5.6% | 3.4% | 2.9% |
| **poor appetite** | None | 1.9% | 6.1% | 9.9% | 9.1% |
|  | Mild | 8.2% | 16.3% | 20.7% | 19.9% |
|  | Moderate | 50.2% | 54.9% | 53.2% | 53.6% |
|  | Overwhelming | 7.3% | 2.7% | 1.6% | 1.8% |
| **restless legs** | None | 2.6% | 5.3% | 8.3% | 10.1% |
|  | Mild | 6.7% | 10.8% | 13.9% | 15.5% |
|  | Moderate | 34.0% | 40.1% | 42.6% | 43.1% |
|  | Overwhelming | 12.4% | 6.9% | 4.4% | 3.5% |
| **Vomiting** | None | 3.1% | 14.3% | 28.0% | 32.4% |
|  | Mild | 10.0% | 23.2% | 28..5% | 29.0% |
|  | Moderate | 45.8% | 47.2% | 36.9% | 33.5% |
|  | Overwhelming | 6.0% | 0.9% | 0.2% | 0.2% |
| **Nausea** | None | 3.9% | 14.5% | 27.2% | 33.0% |
|  | Mild | 5.7% | 12.9% | 16.9% | 17.5% |
|  | Moderate | 33.4% | 42.8% | 39.6% | 36.7% |
|  | Overwhelming | 5.2% | 1.0% | 0.3% | 0.2% |
| **constipation** | None | 3.8% | 9.9% | 15.9% | 17.6% |
|  | Mild | 7.8% | 13.9% | 17.6% | 18.4% |
|  | Moderate | 41.7% | 47.6% | 46.8% | 46.1% |
|  | Overwhelming | 5.6% | 1.8% | 0.9% | 0.7% |
| **diarrhoea** | None | 2.9% | 11.7% | 20.4% | 20.0% |
|  | Mild | 7.5% | 17.0% | 21.6% | 21.5% |
|  | Moderate | 33.4% | 41.6% | 39.3% | 39.4% |
|  | Overwhelming | 15.6% | 4.4% | 2.0% | 2.0% |
| **Sore mouth** | None | 3.6% | 12.5% | 20.0% | 17.9% |
|  | Mild | 8.9% | 18.4% | 22.4% | 21.5% |
|  | Moderate | 41.7% | 46.7% | 43.2% | 44.2% |
|  | Overwhelming | 11.0%% | 3.0% | 1.4% | 1.8% |
| **pruritis** | None | 3.1% | 9.0% | 15.8% | 19.2% |
|  | Mild | 7.5% | 14.4% | 19.1% | 20.7% |
|  | Moderate | 33.0% | 40.6% | 40.8% | 39.8% |
|  | Overwhelming | 17.2% | 7.1% | 3.6% | 2.6% |
