# Supplementary material for: The trajectory of a range of commonly captured symptoms with standard care in people with kidney failure receiving haemodialysis: consideration for clinical trial design
Source: BMC Nephrol. 2023 Nov 17;24:341. doi: 10.1186/s12882-023-03394-w (PMC10656962; doi:10.1186/s12882-023-03394-w)
Supplement: Supplementary file 9 — Additional file 9. Proportions /probabilities of people with moderate or worse severity at baseline moving in and out of severe group over 6 months. [file 12882_2023_3394_MOESM9_ESM.docx]

**Additional file 9: Proportions /probabilities of people with moderate or worse severity at baseline moving in and out of severe group over 6 months**

| **Group** | **Symptoms** | **Prevalence of Severe category at time point 0** | **Proportions of people (those at time point 0) who remained in the severe category at time point 1** | **Proportions of people Moving out of severe category to other categories (moving out from timepoint 0 to 1)** | **Moving into severe category from other categories (moving into timepoint 1 from 0)** | **Difference between moving in and out** |
| --- | --- | --- | --- | --- | --- | --- |
| **≦20% change** | **Difficult sleeping** | 29.8% (111/373) | 44.1% (49/111) | 55.9% (62/111) | 55.9% (62/111) | 0% |
|  | **Weakness** | 28% (134/478) | 35.8% (48/134) | 64.2% (86/134) | 59.7% (71/119) | 4.5% |
|  | **Anxiety** | 16.0% (34/207) | 38.2% (13/34) | 61.8% (21/34) | 62.0% (22/35) | 0.2% |
|  | **Depression** | 20.6% (36/175) | 22.2% (8/36) | 77.8% (28/36) | 77.1% | 0.7% |
|  | **Shortness of breath** | 21.1% (56/266) | 42.9% (24/56) | 57.1% (32/56) | 52.0% (26/50) | 5.1% |
|  | **Poor mobility** | 35.3% (150/425) | 48.0% (72/150) | 52.0% (78/150) | 47.1% (64/136) | 4.9% |
|  | **Changes in skin** | 15.7% (37/235) | 21.62% (8/37) | 78.4% (29/37) | 74.2% (23/31) | 4.2% |
|  | **Drowsiness** | 16.6% (54/326) | 27.8 (15/54) | 72.2% (39/54) | 74.1% (43/58) | -1.9% |
|  | **Pain** | 27.8% (93/334) | 36.6% (34/93) | 63.4% (59/93) | 60.0% (51/85) | 3.4% |
|  | **Poor appetite** | 18.0% (42/233) | 35.7% (15/42) | 64.3% (27/42) | 60.5% (23/38) | 3.8% |
|  | **Restless legs** | 30.9% (97/314) | 43.3% (42/97) | 56.7% (55/97) | 48.8% (40/82) | 7.8% |
| **>20% change** | **Vomiting** | 18% (16/86) | 25% (4/16) | 75% (12/16) | 63.6% (7/11) | 11.4% |
|  | **Nausea** | 28.9% (43/149) | 27.9% (12/43) | 72.1% (31/43) | 62.5% (20/32) | 9.6% |
|  | **Constipation** | 23% (31/135) | 45.2% (14/31) | 54.8% (17/31) | 44.0% (11/25) | 10.8% |
|  | **Diarrhoea** | 22.6% (24/106) | 25% (6/24) | 75.0% (12/24) | 57.1% (8/14) | 17.9% |
|  | **Sore mouth** | 20.6% (44/214) | 22.7% (10/44) | 77.3% (34/44) | 68.8% (22/32) | 8.5% |
|  | **Pruritis** | 27.9% (92/330) | 27.2% (25/92) | 72.8% (67/92) | 63.8% (44/69) | 9.0% |

**(Additional file 10 shows an example of estimating transition probabilities of people reporting feeling anxious at time point 0 and 1).**

**≦ or >20% change estimates was used for ease of interpretation as well as the distribution of the variable as there is natural break around 20% in the data**
