# Supplementary figures and images for: The trajectory of a range of commonly captured symptoms with standard care in people with kidney failure receiving haemodialysis: consideration for clinical trial design
Source: BMC Nephrol. 2023 Nov 17;24:341. doi: 10.1186/s12882-023-03394-w (PMC10656962; doi:10.1186/s12882-023-03394-w)

**Additional File 9: Proportion of change in symptom (feeling anxious) over 6 months period**


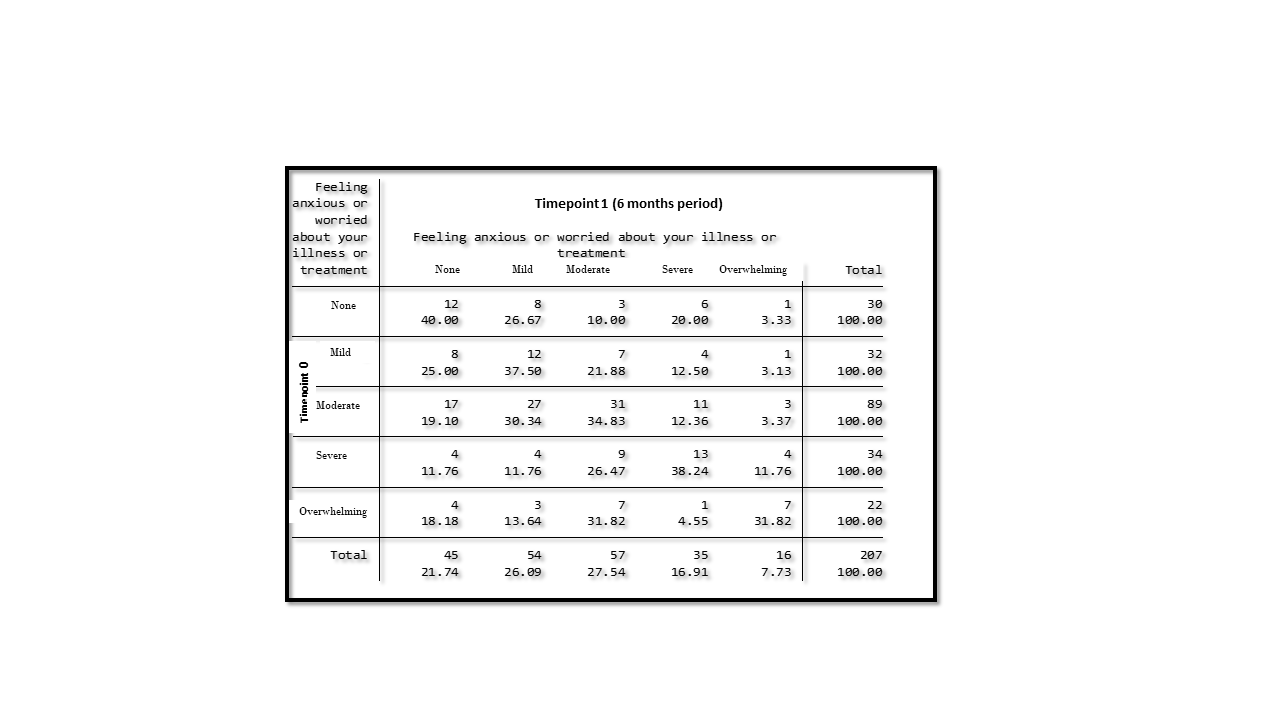

Supplement: Supplementary file 10 — Additional file 10. Proportion of change in symptom (feeling anxious) over 6 months period. [file 12882_2023_3394_MOESM10_ESM.docx]

**Additional File 11: Example of symptom trajectory of feeling anxious (none or mild at baseline)**


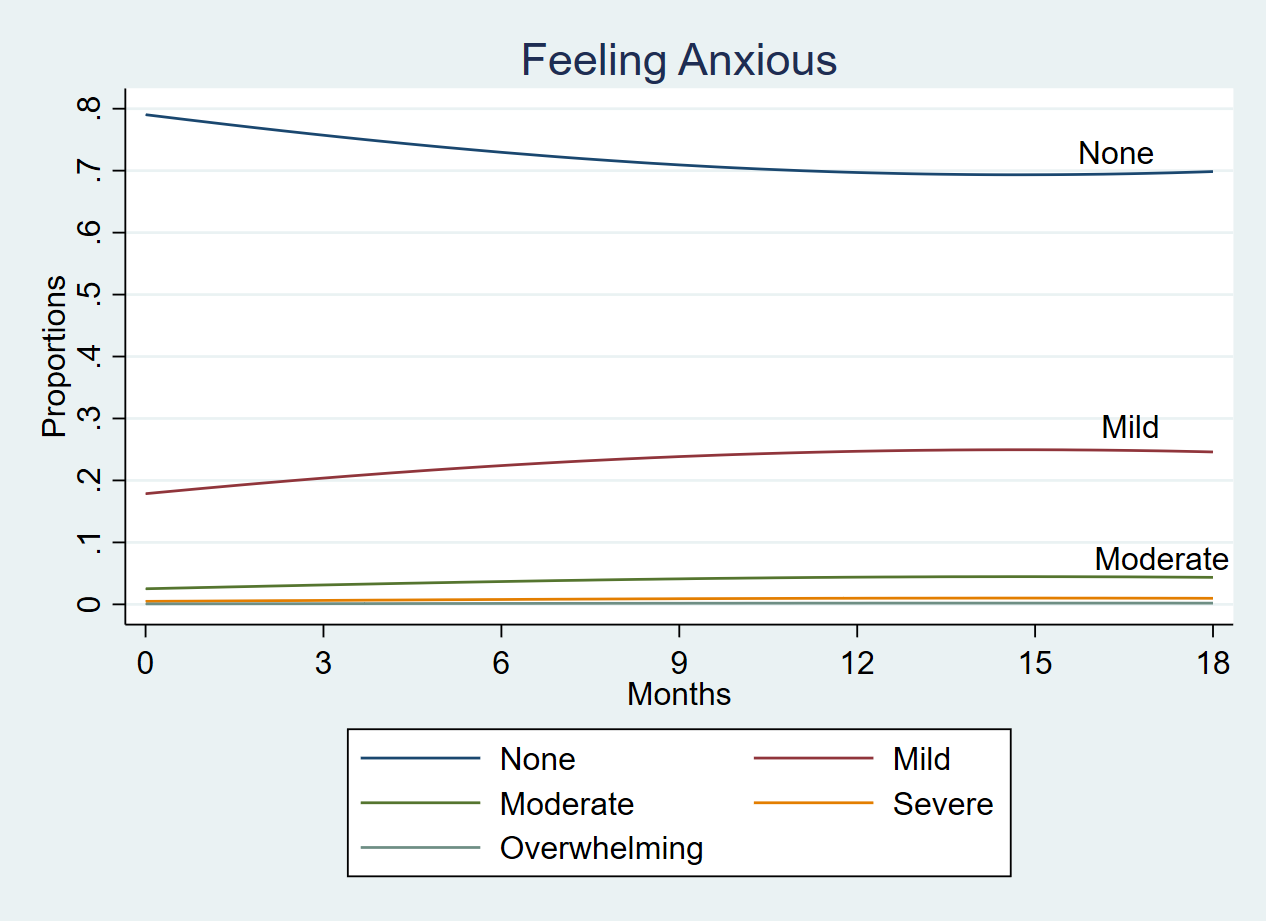

Supplement: Supplementary file 12 — Additional file 12. Example of symptom trajectory of feeling anxious (none or mild at baseline). [file 12882_2023_3394_MOESM12_ESM.docx]
