# Supplementary material for: The trajectory of a range of commonly captured symptoms with standard care in people with kidney failure receiving haemodialysis: consideration for clinical trial design
Source: BMC Nephrol. 2023 Nov 17;24:341. doi: 10.1186/s12882-023-03394-w (PMC10656962; doi:10.1186/s12882-023-03394-w)

**Additional File 10: Example of symptom trajectory of feeling anxious (all degrees of severity at baseline)**


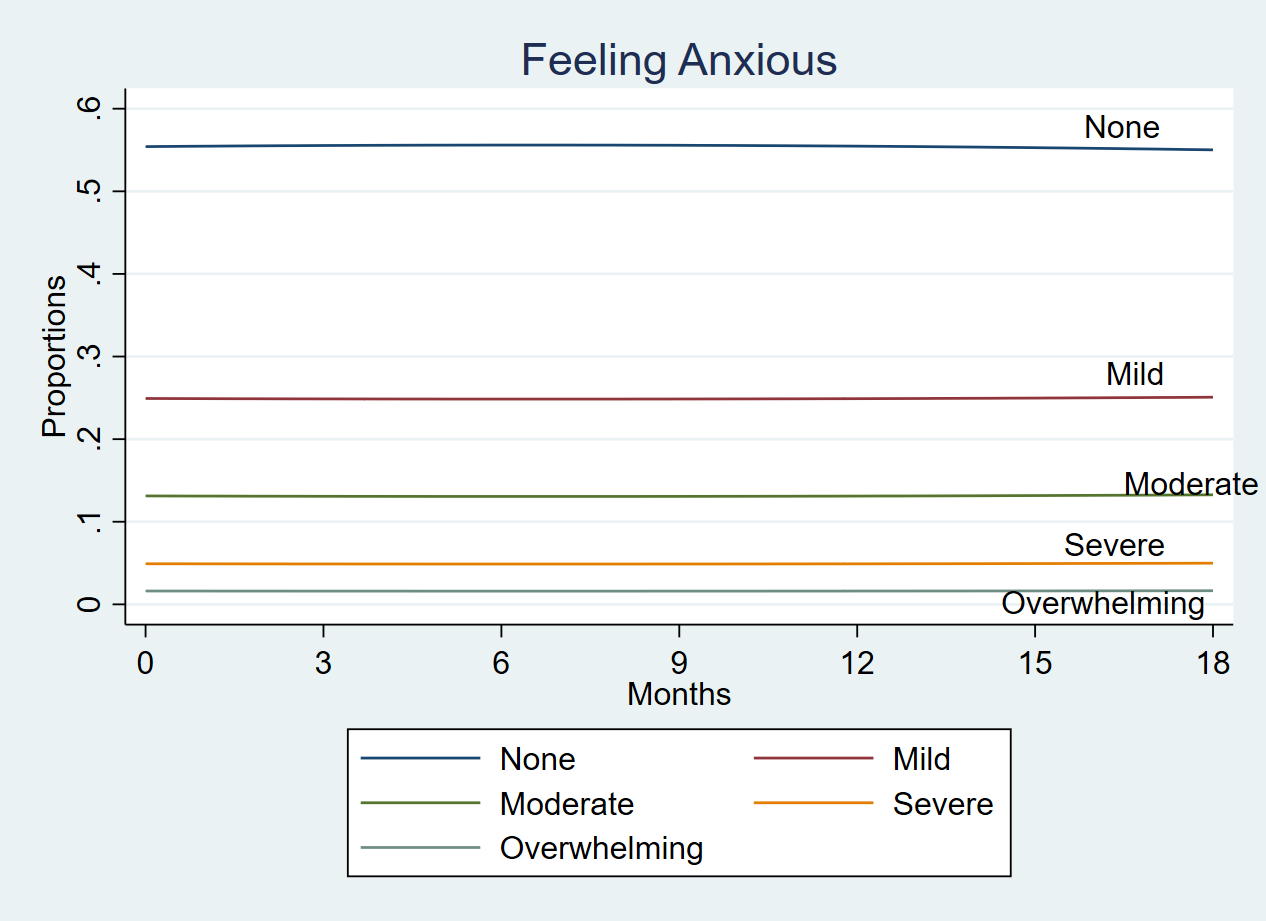

Supplement: Supplementary file 11 — Additional file 11. Example of symptom trajectory of feeling anxious (all degrees of severity at baseline). [file 12882_2023_3394_MOESM11_ESM.docx]
